# Phytostabilization potential and tolerance mechanisms of native species from the Pampa biome in vineyard soil with high levels of Cu, Zn and Mn

**DOI:** 10.1007/s11356-026-37426-3

**Published:** 2026-02-02

**Authors:** Letícia Morsch, Anderson Cesar Ramos Marques, Edicarla Trentin, Talita Andreolli, Filipe Nunes de Oliveira, Matheus Martins Ferreira, Jean Michel Moura-Bueno, Douglas Luiz Grando, Adriele Tassinari, Paola Daiane Welter, Luciane Almeri Tabaldi, Jucinei José Comin, Arcângelo Loss, Cledimar Rogério Lourenzi, Gustavo Brunetto

**Affiliations:** 1 Department of Rural Engineering, Universidade Federal de Santa Catarina (UFSC), Florianópolis, SC Brazil; 2 Department of Soil, Universidade Federal de Santa Maria (UFSM), Santa Maria, RS Brazil; 3 Department of Education, Instituto Federal de Rondônia (IFRO) and Centro Universitário Faema (UNIFAEMA), Ariquemes, RO Brazil

**Keywords:** Excess elements in soils, Phytotoxicity, Phytoremediation, Native species, Grassland, Viticulture

## Abstract

Fungicides applied to grapevines can increase the levels of copper (Cu), zinc (Zn), and manganese (Mn) in soils and cause phytotoxicity to plants. However, plants native to the Pampa biome might have the potential to phytostabilize these metals. The study aimed to (a) verify whether higher levels of Cu, Zn, and Mn in the soil increase the concentrations of these elements in different organs of the native species; (b) determine which variable is most directly associated with biomass variation in the evaluated species; and (c) identify the tolerance mechanisms used by these species to tolerate high levels of Cu, Zn, and Mn in the soil, as well as their phytostabilization potential. To this end, three native species, *Axonopus compressus*, *Paspalum notatum*, and *Paspalum plicatulum*, were grown in vineyard soil and native field soil. The cell wall and vacuole played an important role in detoxifying the metals. The concentrations of photosynthetic pigments were lower in the *A. compressus* and *P. notatum* species grown in the vineyard soil. Metals caused oxidative stress in roots, and the activities of the antioxidant enzymes SOD and POD increased in leaves and roots. The three species showed the lowest dry mass yields in the aerial part. The species *P. notatum* and *P. plicatulum* are the most suitable to be used for the phytostabilization of Cu, Zn, and Mn in vineyards in the Pampa biome.

**Supplementary Information:**

The online version contains supplementary material available at 10.1007/s11356-026-37426-3.

## Introduction

The Pampa biome is located in South America and has a great biodiversity of grassland vegetation (Andrade et al. 2023). Viticulture is an agricultural activity in this biome that does not drastically alter the landscape, causing less environmental impact compared to other agricultural activities (Chomenko and Bencke 2016). As the vines are trellised (Sarmento 2016), this allows light to enter and native species of the Pampa biome to remain between the rows. However, phytosanitary treatments carried out in viticulture can increase the levels of heavy metals such as copper (Cu), zinc (Zn), and manganese (Mn) in the soil, which are observed in several countries around the world (Komárek et al. 2010; Morsch et al. 2024; Beygi & Jalali 2019; Campillo-Cora et al. 2019; Sonoda et al. 2019).

Although Cu, Zn, and Mn are essential for plant development, high levels of these metals in the tissue can cause plant toxicity (Kabata-Pendias 2011; Marschner 2012). In the southern region of Brazil, soils with levels higher than 0.4 mg Cu per dm^−3^ of soil (Mehlich-1), 0.5 mg Zn per dm^−3^ of soil (Mehlich-1), and 0.5 mg Mn per dm^−3^ of soil (KCl 1 mol L^−1^) are considered high for most crops (CQFS-RS/SC 2016). The effects of toxicity caused by Cu, Zn, and Mn can be observed through biochemical and physiological changes in plants (Xiao et al. 2020; Yadav et al. 2021; Schwalbert et al. 2022). Excess heavy metals can also cause nutritional imbalances (Kabata-Pendias 2011; Marschner 2012) and decrease the concentration of photosynthetic pigments in plants, which can reduce the photosynthetic rate (Silva et al. 2022; Marques et al. 2023). They can also cause oxidative stress through the formation of reactive oxygen species (ROS), such as hydrogen peroxide (H_2_O_2_) (Silva et al. 2022). Thus, all these changes in plant metabolism can cause lower growth and biomass production (Silva et al. 2022; Marques et al. 2023) and, in more extreme cases, even plant death (Silva et al. 2022).

Faced with the stress caused by excess metals, the most tolerant plants can use different tolerance mechanisms to reduce metal toxicity, absorption, and mobility. Plants grown with high concentrations of metals, such as Cu, Zn, and Mn, can decrease the translocation of these metals to the shoot and prevent high concentrations of metals from reaching mainly the leaves, where the effects can be more harmful (He et al. 2022; Marques et al. 2023). But also, to maintain adequate cell metabolism, plants can store excess Cu, Zn, and Mn in metabolically less active areas, such as the cell wall and vacuole (Alejandro et al. 2020; Bashir et al. 2021; He et al. 2022; Xiao et al. 2020). In addition, to reduce oxidative stress caused by excess metals, plants can increase the activity of antioxidant enzymes, such as superoxide dismutase (SOD) and guaiacol peroxidase (POD) (Balafrej et al. 2020; Xiao et al. 2020; Kumar et al. 2021; Marques et al. 2023).

Maintaining the native species of the Pampa biome plays important roles in these areas, such as physical protection of the soil and nutrient cycling (Mendes et al. 2025). This is because the soil in these areas is characterized by low levels of clay, organic matter, and cation exchange capacity (CEC) (Silva et al. 2022). In addition, these species can reduce the availability of Cu, Zn, and Mn to grapevines by stabilizing them in the rhizosphere (Schwalbert et al. 2022; Thiesen et al. 2023). Typically, phytoextraction and phytostabilization processes are the most commonly used for the phytoremediation of soils with high levels of heavy metals (Garbisu and Alkorta 2003; Pinto et al. 2016). However, given the physical and chemical characteristics of the soils in the Pampa biome region, the phytostabilization process may be the most suitable (Teodoro et al. 2020), since, unlike phytoextraction, in phytostabilization the plants are not harvested from the area (Yan et al. 2020).

In order for phytostabilization to be more efficient at reducing bioavailable metal levels, it is necessary to select suitable species that are effective and do not cause damage to the ecosystem (Wei et al. 2021). In this sense, local species may be more suitable for use in phytostabilization, in order to achieve the greatest growth and also the development of tolerance mechanisms (Wei et al. 2021). In addition, the species that remain in the areas are the result of environmental pressure to select tolerance mechanisms to favor the permanence of these species in the contaminated areas (Matanzas et al. 2021). For this reason, species native to the biome should preferably be used, as alien species can, for example, through root exudates, modify the chemistry of the rhizospheric soil, the interactions between plants and microorganisms and thus alter the functions of the ecosystem (Weidenhamer and Callaway 2010). Native species of the Pampa biome *Paspalum plicatulum* and *Paspalum notatum* have a contribution of biomass in vineyards (Silva et al. 2020; Morsch et al. 2024), while species of the genus *Axonopus* sp. have a greater contribution of biomass in native field areas (Silva et al. 2020, 2022; Morsch et al. 2024).

Knowing which native species can tolerate high levels of metals is important in order to select possible candidates for phytostabilization, or even to be used as strategic species in future restorations of these areas. In addition, knowing the tolerance mechanisms used by these plants can help in the selection of more suitable species, and these mechanisms can be used in genetic improvement programs in the future (Bashir et al. 2021). The study aimed to (a) verify whether higher levels of Cu, Zn, and Mn in the soil increase the concentrations of these elements in different organs of the native species of the Pampa biome, *Axonopus compressus* (Sw.) P. Beauv.,* Paspalum notatum* Flüggé, and *Paspalum plicatulum* Michx., (b) determine which variable is most directly associated with biomass variation in the evaluated species, and (c) verify the tolerance mechanisms used by these native species of the Pampa biome to tolerate high levels of Cu, Zn, and Mn in the soil, as well as their phytostabilization potential.

## Material and methods

### Description of the experiment

The experiment was conducted in a greenhouse from February to June 2022. The soil used in the experiment was collected from a commercial vineyard (VN) in the municipality of Santana do Livramento, in the state of Rio Grande do Sul, Brazil. The vineyard was planted in 1979 and had high levels of Cu, Zn, and Mn (Table 1). For comparison, soil was collected from an area adjacent to the vineyard, which was under natural grassland or native field (NF) and had not been subject to agricultural cultivation. The soil at both sites was classified as *Typic Hapludalf* (Soil Survey Staff 2006). The physical and chemical characteristics of the soil are shown in Table 1. Three species from the Poaceae family and native to the Pampa biome were used: *P. plicatulum*, *P. notatum*, and *A. compressus.* The experiment lasted for 110 days and was conducted using a completely randomized design with four replications per treatment. To ensure the experiment could be conducted over this period, 10 mL of nutrient solution (Hoagland and Arnon 1950) was added twice per replication, without the addition of Cu, Zn, and Mn, at 50% strength and pH 5.5. The first application was made at 45 days and the second at 58 days after planting the seedlings.

**Table 1:** Initial characterization of the soil collected in the 0–0.20 m layer

	Natural field	Vineyard
Clay (g kg^−1^)	63.55	65.41
Sand (g kg^−1^)	868.74	855.68
Soil organic matter (%)	1.30	0.82
pH in H_2_O (relation 1:1)	5.12	5.67
Available P by Mehlich-1 (mg dm^−3^)	10.17	25.21
Available K by Mehlich-1 (mg dm^−3^)	403.00	448.00
Exchangeable Ca (mg dm^−3^)	245.36	440.32
Exchangeable Mg (mg dm^−3^)	27.09	54.10
Fe (extracted by EDTA) (mg dm^−3^	124.57	90.06
Cu (extracted by Mehlich-1) (mg dm^−3^)	3.29	42.60
Cu (extracted by EDTA) (mg dm^−3^)	2.82	39.20
Zn (extracted by Mehlich-1) (mg dm^−3^)	3.12	17.37
Zn (extracted by EDTA) (mg dm^−3^)	2.19	13.51
Mn (extracted by Mehlich-1) (mg dm^−3^)	55.29	162.54
Mn (extracted by EDTA) (mg dm^−3^)	25.97	91.38
CEC (pH_7.0_) (cmol_c_ kg^−1^)	6.75	5.42
Base saturation (%)	36.89	70.11
Al^3+^ saturation (%)	10.7	0.0

### Rhizobox and soil conditioning

The soil was collected from two areas, one in the inter-row of the VN with 44 years of cultivation and the second in a NF area. The soil samples collected were undeformed to preserve the soil layers and keep them as close as possible to real field conditions. A metal “form” with the dimensions for each replication was used for collection. A trench was dug in each area, and soil was collected using the dimensions of the form within the trench. The form was pressed into the soil (Supplementary material, Figure S1a). The soil was then removed from the trench (Supplementary material, Figure S1b) and placed into the rhizobox (Supplementary material, Figure S1c). After arranging the samples from the two replicates in each rhizobox, the soil was sealed with acrylic and screws (Supplementary material, Figure S1d). The rhizoboxes were made of wood and acrylic, with dimensions of 0.50 m long, 0.45 m high, and 0.045 m deep. Each rhizobox was divided in half to provide two replicates, resulting in each replication having dimensions of 0.25 × 0.45 × 0.045 m.

In the greenhouse, the rhizoboxes were placed on supports inclined at 45°. They were then covered with dark plastic for 75 days to prevent light from entering and to stop the plants from emerging and surviving. After this period, the plastic was removed, and both sides of each rhizobox were covered with aluminum foil, a procedure that continued throughout the experiment to block light (Supplementary material, Figures S2a-b). Every 30 days, the rhizoboxes were rotated so that the lower side replaced the upper side, ensuring better distribution of the roots.

### Collecting, multiplying, and transplanting seedlings

Seedlings of the three species were collected from vineyards and adjacent areas, with all seedlings from each species sourced from the same place. The plants were washed, and each tiller was separated, with both shoot and root systems standardized to 6 cm. The seedlings were transplanted into 15 L plastic trays filled with sand as a substrate and provided with the nutrient solution, following the method suggested by Marques et al. (2020). After 30 days of growth, the seedlings were removed from the sand, and each tiller was washed with distilled water. For the experiment, each tiller was kept with three roots, and both shoot and root lengths were standardized to 6 cm. Five tillers were planted in each experimental replication.

### Tissue dry mass and nutritional composition

At 110 days after planting the seedlings, the plants were collected and separated into leaves, stems, and roots. The senescent leaves were also collected separately and considered as senescent material. The plant parts were cleaned in distilled water and dried in a forced ventilation oven at 65 °C until reaching a constant weight. Dry mass was measured using a precision scale.

The dried samples of leaves, stem, and root samples were ground in a Wiley mill, prepared, and subjected to nitroperchloric digestion (EMBRAPA 2009). The concentrations of Ca, Mg, Cu, Zn, Mn, and Fe in the extracts were determined using an atomic absorption spectrophotometer (AAnalyst 200, Perkin-Elmer, USA). The P concentration was determined according to Murphy and Riley (1962), using a UV–visible spectrophotometer at 882 nm (SF325NM, Bel Engineering, Italy), while the K concentration was quantified using a flame spectrophotometer (Digimed, DM-62, Brazil).

### Cu, Zn, and Mn accumulation, translocation factor (TF), bioconcentration factor (BCF), and tolerance index (TI)

The amounts of Cu, Zn, and Mn accumulated in *P. plicatulum*, *P. notatum*, and *A. compressus* were calculated by multiplying the metal concentrations in leaves, stems, and roots by their corresponding dry masses. For plants growing in VN soil, three indices were determined: the translocation factor (TF), which is the ratio of metal concentrations in the shoots to those in the roots, assessing the species’ ability to translocate Cu, Zn, and Mn; the bioconcentration factor (BCF), which is the ratio of metal concentration in the roots to the concentration of the metal available in the soil; and the tolerance index (TI), which is the ratio of the dry mass of whole plants grown in VN soil to those grown in NF soil. These indices were used to evaluate the tolerance of the three species to high levels of Cu, Zn, and Mn.

### Subcellular fractionation of Cu, Zn, and Mn in leaves and roots

At 110 days after planting the seedlings, leaf and root samples from three species were collected and immediately frozen in liquid N_2_ for subcellular fractionation of Cu, Zn, and Mn. Following the protocol of Huang et al. (2016) with adaptations, 1 g of fresh leaf tissue and 1 g of roots were homogenized in 10 mL of buffer solution containing 50 mM Tris—HCl (pH 7.5), 250 mM sucrose, and 1.0 mM dithioerythritol. The homogenate was centrifuged at 300 × *g* for 5 min at 4° C, yielding a *pellet* that represented the cell wall. The supernatant was transferred to a new tube and centrifuged at 5000 ×*g* for 20 min to obtain the nuclei and plastids’ fraction. The remaining supernatant was centrifuged at 15,000 ×*g* for 30 min to isolate the mitochondrial fraction, while the final supernatant was classified as the soluble fraction. All procedures were conducted at 4 °C. The fractions were dried at 70 °C until reaching a constant weight and then subjected to nitroperchloric digestion (3:1, v/v). The concentrations of Cu, Zn, and Mn in the different fractions were analyzed using an atomic absorption spectrophotometer (AAnalyst 200, Perkin-Elmer, USA).

### Pigments, oxidative stress, and antioxidant enzyme activity

At 110 days after planting the seedlings, leaf and root samples were taken. The roots were washed with distilled water to remove the soil. The leaf and root samples were immediately placed in liquid N_2_ and then stored in an ultrafreezer at −80 °C until the analyses were carried out. Leaf pigments (chlorophyll *a*, *b*, total (a + b) and carotenoids), hydrogen peroxide concentration (H_2_O_2_), and the activity of the enzymes superoxide dismutase (SOD) and guaiacol peroxidase (POD) in leaves and roots were determined.

To quantify pigments, leaves were macerated in liquid N_2_, and then 0.05 g of tissue was incubated with dimethyl sulfoxide (DMSO) at 65 °C until bleached (Hiscox and Israelstam 1979). Absorbance was measured at 470, 645, and 663 nm, with concentrations determined using the Lichtenthaler formula (Lichtenthaler 1987). The concentration of hydrogen peroxide (H_2_O_2_) was determined according to Loreto and Velikova (2001), with samples homogenized in 1.5 mL of 0.1% (w/v) TCA and centrifuged. The concentration of H_2_O_2_ was quantified using a standard calibration curve at 390 nm.

The activity of the SOD enzyme was measured according to the method described by Loreto and Velikova (2001), using a tissue homogenate in 3 mL of 0.05 M sodium phosphate buffer (pH 7.8), supplemented with 1 mM EDTA and 1% Triton X-100. One unit of SOD is defined as the amount of enzyme that inhibits the photoreduction of Nitroblue tetrazolium (NBT) by 50% (Beauchamp and Fridovich 1971). SOD activity was assessed using the colorimetric method established by Giannopolitis and Ries (1977). POD activity was determined following Zeraik et al. (2008), with a reaction mixture of 1.0 mL of potassium phosphate buffer (pH 6.5, 100 mmol L^−1^), 1.0 mL of guaiacol (15 mmol L^−1^), 1.0 mL of H_2_O_2_ (3 mmol L^−1^), and 50 µL of the plant extract, monitoring the oxidation of guaiacol by measuring the increase in absorbance at 470 nm.

### Statistical analysis

The subcellular distribution of Cu, Zn, and Mn was analyzed in relation to areas (NF and VN), species (*P. plicatulum*, *P. notatum*, and *A. compressus*), and fractions in a trifactorial design. For other variables, a bifactorial design considering only areas and species was used. Data were analyzed through variance analysis, and means were separated using the Scott-Knott test at a 5% significance level (Scott and Knott 1974). Statistical analyses were performed using the ExpDes.pt package in R software (R Core Team 2021). Additionally, a multiple linear regression model was applied to assess the importance of variables, including metal concentrations in different plant tissues and total plant concentration, in explaining variations in dry mass production. The model’s variable importance was quantified using the “VARIMP” metric in the “caret” package in the R software (Kuhn 2016).

To explore variance and identify complex interactions between variables, principal component analysis (PCA) was applied to a wide range of measurements, including metal concentrations, chlorophyll and carotenoid levels, H_2_O_2_ concentrations, and enzyme activities (SOD and POD) in the leaves and roots of the species. PCA was used to analyze the similarity between the VN and NF areas as well as the species (*A. compressus*, *P. notatum*, and *P. plicatulum*). This approach allowed for the identification of complex interactions between the variables and the comparison of the VN and NF areas, as well as the species. The analysis was performed using the “FactoMineR” and “factoextra” R packages (R Core Team 2021).

## Results

### Dry mass, Cu, Zn, and Mn accumulated in organs and indices

**Fig. 1:**
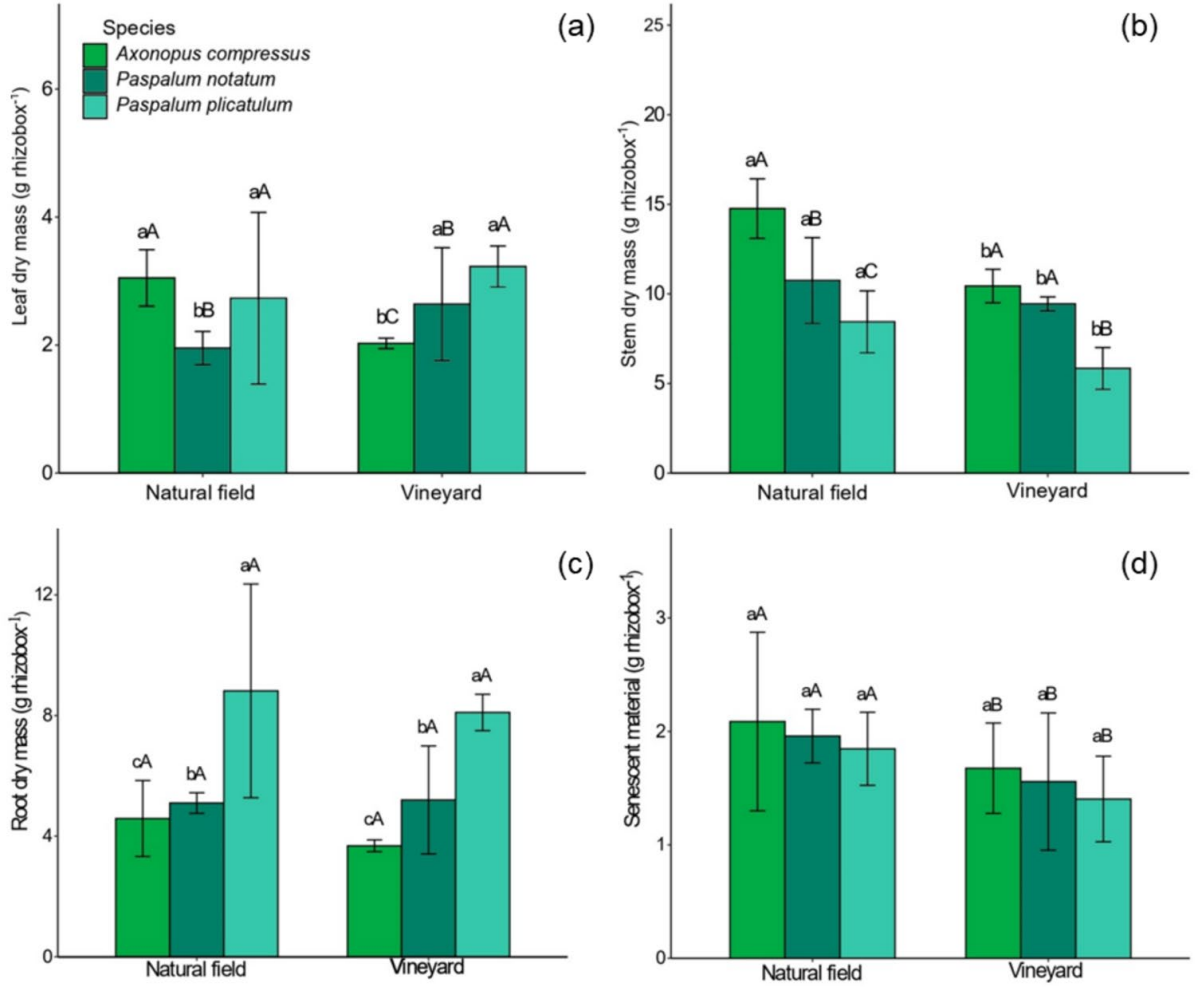
Dry mass production of leaves (**a**), stems (**b**), roots (**c**), and senescent material (**d**) of *A. compressus*, *P. notatum*, and *P. plicatulum*, grown in native field and vineyard soil. The capital letters compare species within each area and lower case letters compare species between areas. Bars with equal letters do not differ statistically by the Scott-Knott test at 5% (*P* < 0.05).

When grown in VN soil, *A. compressus* showed the lowest leaf dry mass, while *P. notatum* exhibited the highest, compared to NF soil (Fig. 1a). In NF soil, however, *P. notatum* produced less leaf dry mass than the other species. In VN soil, *P. plicatulum* presented the highest leaf dry mass, whereas *A. compressus* remained the lowest (Fig. 1a). Reductions in stem dry mass were observed for all species in VN soil compared to NF soil, with decreases of 29.3% for *A. compressus*, 12.1% for *P. notatum*, and 30.7% for *P. plicatulum* (Fig. 1b). Among them, *A. compressus* had the highest stem dry mass in NF soil, while *P. plicatulum* had the lowest in both soil types. Regarding root dry mass, *P. plicatulum* had the highest values and *A. compressus* the lowest across both NF and VN soils (Fig. 1c). For senescent material, all three species showed lower dry mass when grown in the VN soil, compared to NF (Fig. 1d).

**Fig. 2:**
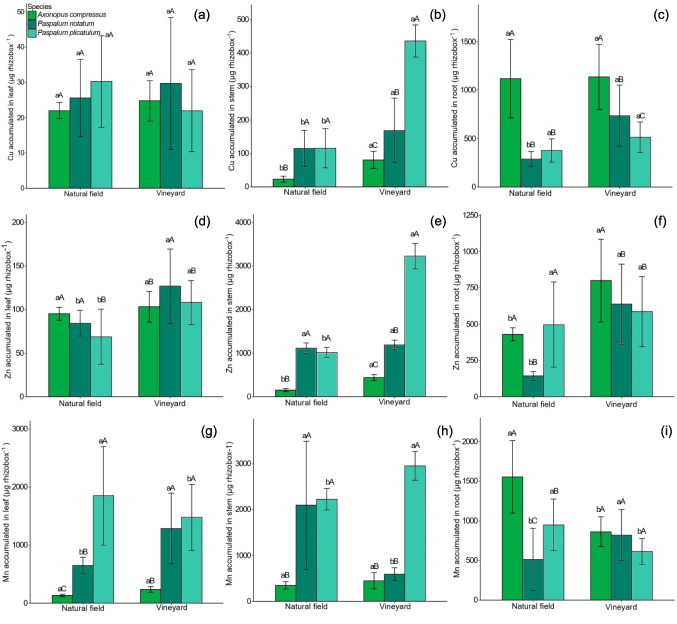
Cu accumulated in leaves (**a**), stem (**b**), and roots (**c**); Zn accumulated in leaves (**d**), stem (**e**), and roots (**f**); and Mn accumulated in leaves (**g**), stem (**h**), and roots (**i**) of *A. compressus*, *P. notatum*, and *P. plicatulum*, grown in native field and vineyard soil. The capital letters compare the species within each area, and the lower case letters compare the species between the areas. Bars with equal letters do not differ statistically by the Scott-Knott test at 5% (*P* < 0.05)

The amount of Cu accumulated in the leaves did not differ among the three species when grown in NF and VN soils (Fig. 2a). In the stem, Cu accumulation increased under VN soil conditions with values 2.5, 0.46, and 2.77 times higher for *A. compressus*, *P. notatum*, and *P. plicatulum*, respectively, compared to the NF soil (Fig. 2b). Among the species, *A. compressus* showed the lowest and *P. plicatulum* the highest Cu accumulation in the stem when grown in VN soil. In the roots, *P. notatum* accumulated more Cu under VN soil than under NF soil (Fig. 2c). Under VN conditions, *A. compressus* accumulated the highest and *P. plicatulum* the lowest Cu concentrations in the roots. Total Cu accumulation (leaves, stem, and roots) in *A. compressus* did not differ between soil types and was the highest among the species evaluated (Supplementary material, Table S1). In contrast, *P. notatum* and *P. plicatulum* accumulated 117% and 85% more Cu, respectively, when grown in the VN soil compared to NF soil (Supplementary material, Table S1).

When grown in VN soil, *P. notatum* and *P. plicatulum* accumulated 50% and 57% more Zn in their leaves, respectively, compared to NF soil (Fig. 2d; supplementary material, Figure S3). Among the species, *P. notatum* exhibited the highest Zn accumulation in leaves under VN conditions. In the stem, Zn accumulation increased by 187% in *A. compressus* and 217% in *P. plicatulum* when cultivated in the VN soil compared to NF (Fig. 2e; supplementary material, Figure S3). Under these conditions, *A. compressus* showed the lowest and *P. plicatulum* the highest Zn accumulation in the stem. In the roots, Zn concentrations were 86% and 329% higher in *A. compressus* and *P.* notatum, respectively, when grown in VN soil compared to NF soil (Fig. 2f; supplementary material, Figure S3). *A. compressus* accumulated the highest Zn content in the roots among the species under VN conditions. Considering total Zn accumulation (leaves, stem, and roots), *A. compressus*, *P. notatum*, and *P. plicatulum* accumulated 98%, 46%, and 148% more Zn, respectively, when grown in VN soil compared to NF (Supplementary material, Table S1). Furthermore, total Zn accumulation was higher in VN soil than in NF soil for all evaluated species (Supplementary material, Table S1). Overall, *A. compressus* accumulated the least and *P. plicatulum* the most Zn in the whole plant, regardless of soil type.

In VN, *P. plicatulum* exhibited the highest accumulation of Mn in leaves*,* while *A. compressus* showed the lowest; *A. compressus* accumulated the least Mn in leaves across both NF and VN soils (Fig. 2g). In stems, Mn accumulation was greatest in *P. plicatulum* and lowest in *A. compressus* under VN conditions; *A. compressus* accumulated the least Mn in stems across both NF and VN soils (Fig. 2h). In roots, *P. notatum* accumulated the least Mn in NF soil, while *A. compressus* and *P. plicatulum* consistently exhibited the highest levels (Fig. 2i). When considering total plant Mn accumulation, *P. plicatulum* led in both soils, with *A. compressus* showing the lowest (Supplementary material, Table S1).

**Fig. 3:**
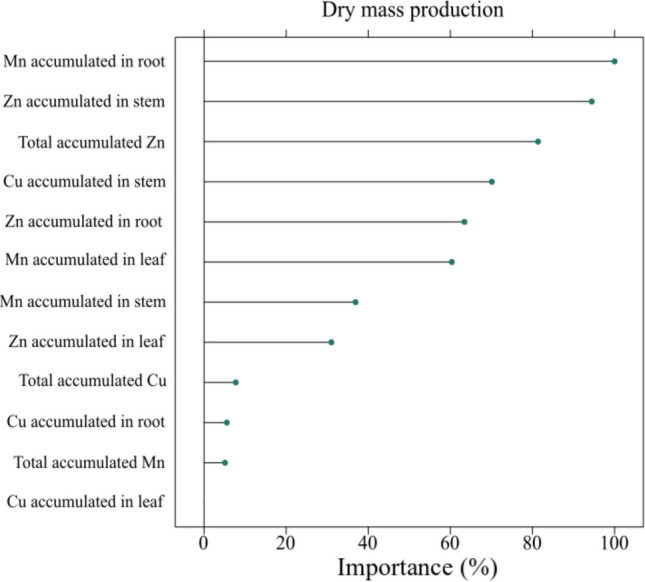
Importance (relative %) of each variable in explaining the variation in dry mass production in *A. compressus*, *P. notatum*, and *P. plicatulum*, grown in native field and vineyard soil

Among the factors explaining dry mass variation in VN and NF soils, Mn accumulation in roots, Zn accumulation in stems, and total Zn uptake were most influential (Fig. 3). All species showed the highest Cu concentration in roots (TF < 1; Supplementary material, Table S3). and *A. compressus* particularly amassed the highest levels of Zn and Mn in roots (TF < 1). Conversely, *P. notatum* translocated more Zn and Mn to shoots, while *P. plicatulum* localized primarily Mn in the aerial part (TF > 1). A TF above 1 indicates preferential metal accumulation in shoots, while values below 1 signify root accumulation. All species displayed BCF > 1.0 for Cu, Zn, and Mn, yet the TI was < 1.0 for all, with *A. compressus* showing the lowest value, indicating lower tolerance under VN conditions.

### Subcellular distribution of Cu, Zn, and Mn in leaves and roots

In the leaves and roots of *A. compressus*, *P. notatum*, and *P. plicatulum*, over 90% of Cu, Zn, and Mn were localized in the cell wall and soluble fraction (Tables 2, 3, and 4; Supplementary Material, Figure S4). No significant differences were observed in the nuclei and plastids or mitochondrial fractions among species or between soils (VN and NF) (Table 2). All three species exhibited higher Cu concentration in the cell wall of leaves and roots when grown in the VN soil compared to NF. Similarly, Cu concentrations in the root soluble fraction were also higher under VN conditions. *A. compressus* had the lowest Cu levels in the leaf cell wall and soluble fraction, but the highest in roots, compared to the other species (Table 2).

**Table 2:** Subcellular Cu fractions in leaves and roots of *P. plicatulum*, *P. notatum*, and *A. compressus*, grown in native field and vineyard soil

Leaf Cu concentration (mg kg^−1^)						
	NF			VN		
Fractions	*A. compressus*	*P. notatum*	*P. plicatulum*	*A. compressus*	*P. notatum*	*P. plicatulum*
Cell wall	0.79 bBα^a^	1.84 bAα	1.42 bAα	1.54 aCα	4.19 aAα	2.73 aBα
Plastids and nucleus	0.05 aAβ	0.14 aAγ	0.13 aAγ	0.21 aAβ	0.52 aAγ	0.14 aAγ
Mitochondria	0.03 aAβ	0.09 aAγ	0.04 aAγ	0.03 aAβ	0.07 aAδ	0.07 aAγ
Soluble	0.50 aBα	1.15 aAβ	0.72 aBβ	0.55 aBβ	1.18 aAβ	0.96 aAβ
Root Cu concentration (mg kg^−1^)						
	NF			VN		
Fractions	*A. compressus*	*P. notatum*	*P. plicatulum*	*A. compressus*	*P. notatum*	*P. plicatulum*
Cell wall	6.72 bAα	4.11 bBα	3.66 bBα	22.57 aAα	8.77 aCα	13.78 aBα
Plastids and nucleus	0.75 aAβ	0.34 aAγ	0.35 aAβ	1.21 aAγ	0.51 aAγ	1.21 aAγ
Mitochondria	0.47 aAβ	0.13 aAγ	0.25 aAβ	0.48 aAγ	0.18 aAγ	0.37 aAγ
Soluble	7.11 bAα	2.96 bBβ	2.69 bBα	13.89 aAβ	5.09 aCβ	7.80 aBβ

^a^Capital letters compare the species within each area and fraction, lower case letters compare the areas within each fraction and species and Greek letters compare the fraction within each area and species. Equal letters do not differ statistically by the Scott-Knott test at 5% (*P* < 0.05)

**Table 3:** Subcellular Zn fractions in leaves and roots of *P. plicatulum*, *P. notatum*, and *A. compressus*, grown in native field and vineyard soil

Leaf Zn concentration (mg kg^−1^)						
	NF			VN		
Fractions	*A. compressus*	*P. notatum*	*P. plicatulum*	*A. compressus*	*P. notatum*	*P. plicatulum*
Cell wall	3.23 bCβ^a^	6.45 bAα	4.04 bBα	5.67 aBα	7.85 aAα	8.17 aAα
Plastids and nucleus	0.24 aAγ	0.40 aAβ	0.37 aAγ	0.35 aAβ	0.50 aAγ	0.79 aAγ
Mitochondria	0.24 aAγ	0.35 aAβ	0.13 aAγ	0.32 aAβ	0.36 aAγ	0.29 aAγ
Soluble	4.82 bBα	6.12 aAα	2.71 bCβ	6.21 aAα	5.94 aAβ	6.12 aAβ
Root Zn concentration (mg kg^−1^)						
	NF			VN		
Fractions	*A. compressus*	*P. notatum*	*P. plicatulum*	*A. compressus*	*P. notatum*	*P. plicatulum*
Cell wall	8.15 bAα	6.12 aBα	5.53 bBα	14.82 aAα	5.05 aBα	15.77 aAα
Plastids and nucleus	1.34 aAγ	0.64 aAγ	0.68 bAγ	1.69 aAγ	0.37 aBβ	2.08 aAγ
Mitochondria	0.52 aAγ	0.15 aAγ	0.26 aAγ	0.53 aAδ	0.28 aAβ	0.51 aAδ
Soluble	3.04 bAβ	2.03 bAβ	2.37 bAβ	7.50 aAβ	5.04 aBα	8.25 aAβ

^a^Capital letters compare the species within each area and fraction, lower case letters compare the areas within each fraction and species and Greek letters compare the fraction within each area and species. Equal letters do not differ statistically by the Scott-Knott test at 5% (*P* < 0.05)

**Table 4:** Subcellular Mn fractions in leaves and roots of *P. plicatulum*, *P. notatum*, and *A. compressus*, grown in native field and vineyard soil

Leaf Mn concentration (mg kg^−1^)						
	NF			VN		
Fractions	*A. compressus*	*P. notatum*	*P. plicatulum*	*A. compressus*	*P. notatum*	*P. plicatulum*
Cell wall	7.76 aCα^a^	40.77 aBα	76.41 aAα	7.31 aBα	10.39 bBα	67.66 bAα
Plastids and nucleus	0.81 aAα	4.59 aAγ	10.16 aAγ	0.39 aBα	1.09 aBα	10.69 aAγ
Mitochondria	0.26 aAα	1.49 aAγ	3.89 aAγ	0.13 aAα	0.31 aAα	3.72 aAγ
Soluble	6.18 aCα	23.99 aBβ	54.71 aAβ	4.45 aBα	5.23 bBα	42.92 bAβ
Root Mn concentration (mg kg^−1^)						
	NF			VN		
Fractions	*A. compressus*	*P. notatum*	*P. plicatulum*	*A. compressus*	*P. notatum*	*P. plicatulum*
Cell wall	43.51 aAα	29.23 aBα	28.97 aBα	33.08 bAα	11.71 bCα	14.38 bBα
Plastids and nucleus	2.45 aAγ	1.52 aAγ	1.40 aAγ	1.53 aAγ	0.51 aAγ	0.87 aAγ
Mitochondria	0.43 aAγ	0.07 aAγ	0.19 aAγ	0.33 aAγ	0.20 aAγ	0.28 aAγ
Soluble	12.62 aAβ	5.20 aBβ	7.27 aBβ	9.70 bAβ	4.41 aBβ	5.68 aBβ

^a^Capital letters compare the species within each area and fraction, lower case letters compare the areas within each fraction and species and Greek letters compare the fraction within each area and species. Equal letters do not differ statistically by the Scott-Knott test at 5% (*P* < 0.05)

Regarding Zn distribution, no differences were found in the nuclei and plastids or mitochondrial fractions of leaves across species or soils (Table 3). However, Zn concentrations in the leaf wall were higher in all species when grown in VN soil. Additionally, *A. compressus* and *P. plicatulum* showed increased Zn levels in the leaf soluble fraction under VN conditions (Table 3). In the roots, both species showed higher Zn concentrations in the cell wall when grown in VN soil, while all three species exhibited greater Zn levels in the soluble fraction. *P. plicatulum* additionally accumulated higher Zn concentrations in the nuclear and plastid fractions of the roots in VN soil compared to NF (Table 3). In contrast, *P. notatum* showed the lowest Zn concentrations in both the cell wall and soluble fraction of roots under VN conditions.

For Mn, *P. notatum* and *P. plicatulum* exhibited lower Mn concentrations in the leaf cell wall and soluble fractions when grown in VN soil compared to NF. Despite this, *P*. *plicatulum* had the highest Mn concentrations in both fractions under both soil types and also accumulated greater Mn in the nuclear and plastid fractions of leaves in VN soil, compared to the other species. Across all three species, Mn concentrations in the nuclear, plastids, and mitochondrial fractions of leaves and roots did not differ significantly between soils. In the roots, all species exhibited reduced Mn concentrations in the cell wall when grown in VN soil. *A. compressus* had the lowest Mn concentrations in the soluble fraction under VN conditions, but showed the highest overall Mn levels in both the cell wall and soluble fraction of the roots (Table 4).

### Nutritional changes

When grown in VN soil compared to NF, *A. compressus* exhibited higher concentrations of P, Ca, and Mg in the leaves; P, K, Ca, and Mg in the stem; and P, K, Ca, Mg, and Fe in the roots (Supplementary material, Table S3). *P. notatum* showed increased concentrations of Ca and Mg and decreased K and Fe in the leaves; higher levels of P, K, and Mg in the stem; and greater concentrations of P, K, Ca, and Mg in the roots in VN soil. Similarly, *P. plicatulum* showed higher K and lower Ca and Mg concentrations in the leaves; increased concentrations of P, Ca, Mg, and Fe in the stem; and higher P, K, and Mg and lower Fe in the roots (Supplementary material, Table S3).

### Pigments, oxidative stress, and antioxidant enzyme activity

**Fig. 4:**
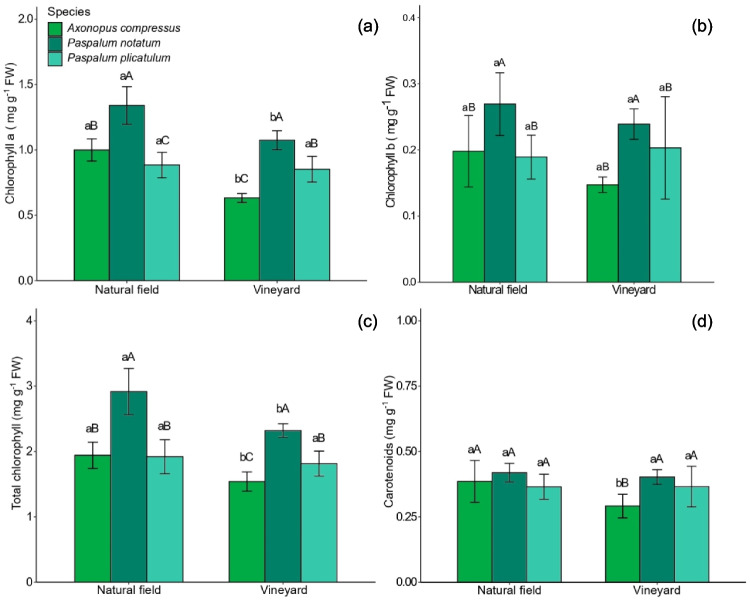
Concentrations of chlorophyll *a* (**a**), chlorophyll *b* (**b**), total chlorophyll (**c**), and carotenoids (**d**) in *A. compressus*, *P. notatum*, and *P. plicatulum*, grown in native field and vineyard soil. The capital letters compare species within each area and lower case letters compare species between areas. Bars with equal letters do not differ statistically by the Scott-Knott test at 5% (*P* < 0.05)

Chlorophyll content and oxidative stress indicators varied among species and soil types. *A. compressus* exhibited the lowest concentrations of chlorophyll *a* (Chla), total chlorophyll (Chl total), and carotenoids when grown in VN soil compared to the NF (Fig. 4a and c–d). Similarly, *P. notatum* showed reduced levels of Chla and total Chl under VN soil conditions (Fig. 4a and c). In contrast, chlorophyll and carotenoid concentrations in *P. plicatulum* were not significantly affected by soil type. Among the species, *P. notatum* displayed the highest concentration of Chla, chlorophyll *b* (Chlb), and total Chl in both VN and NF soils (Fig. 4a–c).

**Fig. 5:**
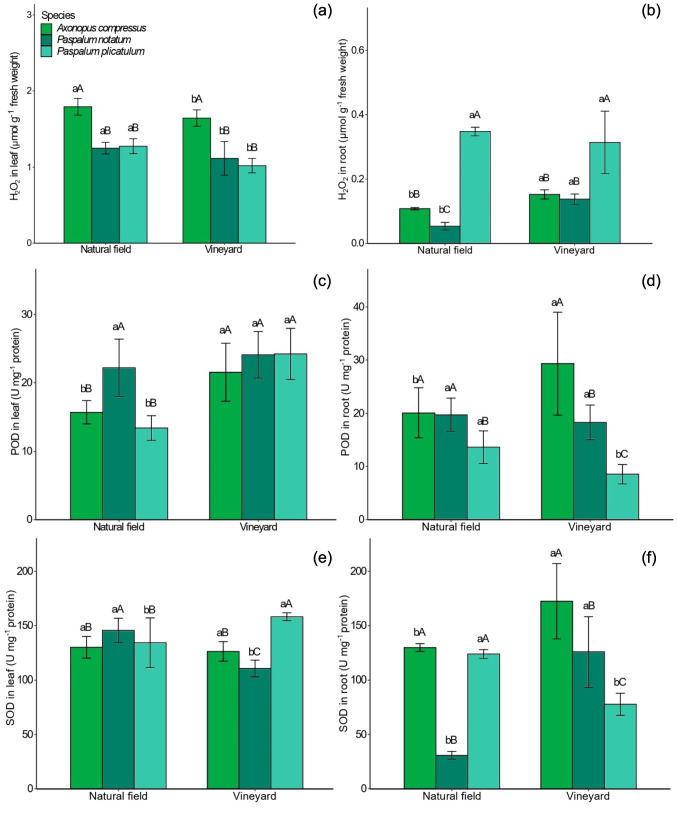
Values of H_2_O_2_ in leaves (**a**) and roots (**b**); activity of the enzymes guaiacol peroxidase (POD) in leaves (**c**) and roots (**d**); and superoxide dismutase (SOD) in leaves (**e**) and roots (**f**) of *A. compressus*, *P. notatum*, and* P. plicatulum*, grown in native field and vineyard soil. The capital letters compare species within each area, and lower case letters compare species between areas. Bars with equal letters do not differ statistically by the Scott-Knott test at 5% (*P* < 0.05)

The levels of H_2_O_2_ in leaves of *A. compressus*,* P. plicatulum*, and *P. notatum* were higher when grown in NF soil (Fig. 5a). *A. compressus* had the highest H_2_O_2_ levels in leaves, while *P. plicatulum* had the highest levels in roots across both soil types (Fig. 5a and b). POD activity in leaves increased under VN conditions for *A. compressus* and *P. plicatulum* (Fig. 5c). In roots, *A. compressus* showed the highest POD activity under VN soil, whereas *P. plicatulum* consistently exhibited the lowest activity across both soils (Fig. 5d). SOD activity in leaves was elevated in *P. plicatulum* and reduced in *P. notatum* under VN conditions (Fig. 5e). Among the species, *P. plicatulum* had the highest and *P. notatum* the lowest leaf SOD activity in VN soil. In roots, *A. compressus* and *P. notatum* exhibited greater SOD activity when grown in VN soil, while *P. plicatulum* showed the opposite trend, with higher activity under NF conditions. Under VN soil, *A. compressus* presented the highest and *P. plicatulum* the lowest SOD activity in roots (Fig. 5f).

### Principal component analysis

**Fig. 6:**
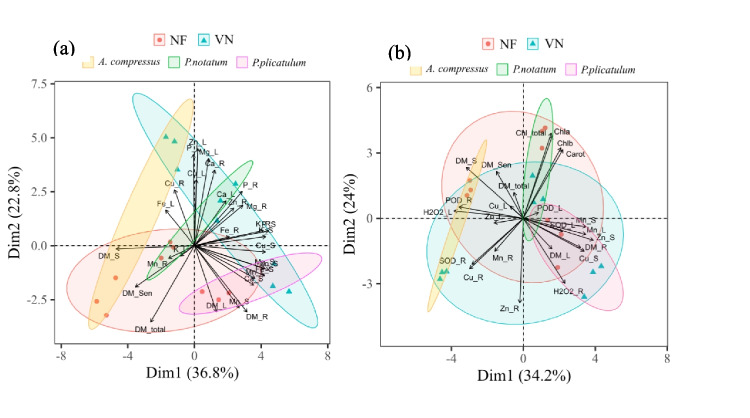
Dry mass in leaves (DM_L), stem (DM_S), roots (DM_R), senescent material (DM_ Sen), and total (DM_total); Cu, Zn, Mn, Fe, P, Ca, Mg, and K concentrations in leaves (Cu_L, Zn_L, Mn_L, Fe_L, P_L, Ca_L, Mg_L, and K_L), stem (Cu_S, Zn_S, Mn_S, Fe_S, P_S, Ca_S, Mg_S, and K_S), and roots (Cu_R, Zn_R, Mn_R, Fe_R, P_R, Ca_R, Mg_R, and K_R); concentration of chlorophyll **a** (Chla), **b** (Chlb), total (Chl_total), and carotenoids (Carot); concentration of H_2_O_2_ in leaves (H2O2 _L) and roots (H2O2 _R); activity of the enzyme SOD in leaves (SOD_L) and roots (SOD_R); activity of the enzyme POD in leaves (POD_L) and roots (POD_R), in the species *A. compressus*,* P. notatum*, and *P. plicatulum*

The analysis showed a split between VN and NF (Fig. 6a). The variables of dry mass in leaves, stem, roots, senescent and total material, concentration of Mn in stem and roots, and K in leaves are related to NF (Fig. 6a). The other metal and nutrient variables in leaves, stems, and roots are related to VN (Fig. 6a). The variable Mn in roots showed an inverse relationship with Zn in roots (Fig. 6a). The relationship between the species and each metal shows that the variables Fe in leaves (Fe_L), Cu in roots (Cu_R), and stem dry mass (DM_S) are related to the species *A. compressus* (Fig. 6a). Ca in leaves (Ca_L), Mn in roots (Mn_R), K in leaves (K_L), and dry mass of senescent material (DM_Sen) are related to the species *P. notatum* (Fig. 6a). The variables of dry mass in leaves (DM_L) and roots (DM_R), Ca, Mg, Fe, Zn, and Mn in the stem (Ca_S, Mg_S, Fe_S, Zn_S, and Mn_S), and Mn in leaves (Mn_L) are related to *P. plicatulum* (Fig. 6a).

The PCA also showed a division between NF and VN, although this division is subtle (Fig. 6b). The concentrations of Cu, Zn, and Mn in roots (Cu_R, Zn_R, and Mn_R) and the activity of the SOD enzyme are inversely related to the concentration of chlorophyll *a* (Chla), *b* (Chlb), total (Chl_total), and carotenoids (Carot) (Fig. 6b). The variables Cu, Zn, and Mn in the stem, dry mass in leaves (DM_L) and roots (DM_R), and H_2_O_2_ in roots, which are associated with *P. plicatulum*, showed an inverse relationship with H_2_O_2_ in leaves, POD_R, DM_S, and DM_sen, which, in turn, are related to *A. compressus* (Fig. 6b).

## Discussion

### Accumulation and subcellular distribution of Cu, Zn, and Mn in *A. compressus*, *P. notatum*, and* P. plicatulum*

*P. notatum* accumulated the highest amounts of Cu, Zn, and Mn in roots when grown in the VN soil (Fig. 2c, f, and i). The highest concentrations of Cu and Zn are usually observed in roots (Gupta et al. 2019). This can be attributed to the low mobility of these metals within the plant (Kabata-Pendias 2011). Cu is strongly bound to the N of proteins and therefore has low mobility in plants (Kabata-Pendias 2011). On the other hand, Zn is bound to light organic compounds in the xylem, fluids, and other plant tissue extracts, which may suggest greater mobility than Cu in plants (Kabata-Pendias 2011). The highest amounts of Mn were accumulated in the shoot of *P. notatum* and *P. plicatulum*, indicating greater mobility of this metal (Shao et al. 2017). The accumulation of metals in roots, under conditions of excess metals in the soil, is also an important tolerance mechanism used to avoid transport to the organs of the shoot, where the effects of metals are more harmful (Yadav et al. 2021; He et al. 2022), since leaves are the most metabolically active organs of the plant. This happens because when metals are inside the cytosol, plants can reduce metal toxicity by complexing ions with organic and inorganic ligands (Yan et al. 2020). In the stem, the accumulated amount of Cu, Zn, and Mn in *P. plicatulum*, Cu and Zn in *A. compressus*, and Cu in *P. notatum* was higher when the plants were grown in the VN soil (Fig. 2b, e, and h). This is attributable to the ability of the stem to retain metals, which reduces transport to the leaves, causing less damage to the photosynthetic apparatus (Marques et al. 2023).

*A. compressus*, *P. notatum*, and *P. plicatulum* showed higher concentrations of Cu and Zn in the cell wall and soluble fraction, which mainly contains vacuoles (Tables 2 and 3). This is a tolerance mechanism used by these species to detoxify metals. Thus, plants maintain optimal cell metabolism under conditions of high concentrations of metals such as Cu, Zn, and Mn (He et al. 2022; Mwamba et al. 2016; Zhang et al. 2019). This is attributable to the presence of polysaccharides and proteins in the cell wall, and their functional groups such as carboxyl, hydroxyl, amino, and aldehyde, when deprotonated, generate negative charges that can adsorb metals, reducing their transport (Fu et al. 2011; Yang et al. 2018; Xiao et al. 2020). In addition, vacuoles are metabolically inactive but play an important role in regulating the distribution of heavy metals (Bashir et al. 2021). This is because, in the vacuole, metals can bind mainly to organic acids, reducing the activity of free ions (Fu et al. 2011; Xiao et al. 2020). The concentrations of Cu, Zn, and Mn in the species evaluated hardly differed in the nucleus, plastid, and mitochondria fractions, where excess metals can be extremely toxic (Bashir et al. 2021). This suggests that the mechanism of retaining ions in the cell wall or storing them in the vacuole was used efficiently in all three species.

The accumulated amount of Mn in the roots of *A. compressus* and *P. plicatulum* was lower when grown in the VN soil. In addition, the concentrations of Mn in the cell wall and soluble fraction in leaves of *P. notatum* and *P. plicatulum* were lower when grown in the VN soil. This was also observed in the cell wall in the roots of the three species and in the soluble fraction of the *A. compressus* species. The result can be attributed to the relationships between the metals Cu, Zn, and Mn, which can be antagonistic and/or synergistic (Kabata-Pendias 2011; Marschner 2012). In addition, the PCA results show that Mn in the root had an inverse relationship with Zn in the root (Fig. 6a). This can be attributed to the similarity of the ionic radii of bivalent cations such as Cu, Zn, Mn, and Fe. Thus, Zn can replace any of these cations and be absorbed by the roots (Kumar et al. 2008). Thus, the high availability of Zn or Mn can strongly decrease the concentration of the other (Marschner 2012). Our results are in line with those of Marastoni et al. (2019), who observed that vines grown with increasing concentrations of Cu showed antagonistic relationships between Zn and Mn.

### Physiological and biochemical changes in *A. compressus*,* P. notatum*, and* P. plicatulum*, grown with high levels of Cu, Zn, and Mn

Shoot dry mass production was lower in *A. compressus*, *P. notatum*, and *P. plicatulum* when grown in the VN soil (Fig. 1a and b). This can be attributed mainly to the amount of Cu, Zn, and Mn accumulated in leaves, stems, and/or roots and, consequently, to the variation in nutritional status (Figs. 2a–i and 3, supplementary material, Tables S3). The reduction in plant dry mass may be associated with the lower adaptability of these plants to metals, since the concentrations of metals in the tissues may have reached toxic levels (Schwalbert et al. 2022; Thiesen et al. 2023). In addition to the toxic effect of metals on the nutritional status of plants, these concentrations in tissues can affect various other biochemical and physiological processes such as cell division, nutrient and water transport in plants, photosynthesis, respiration, oxidative stress, and damage to sensitive organelles, causing a reduction in biomass production (Alsafran et al. 2023). Similar results were observed in species native to South America when grown with high levels of Cu (Marques et al. 2023; Silva et al. 2022) and Zn in the soil (Schwalbert et al. 2022), and Mn in the nutrient solution (Thiesen et al. 2023).

*A. compressus* and *P. notatum* showed lower concentrations of photosynthetic pigments when grown in the VN soil. This reduction in the concentration of photosynthetic pigments may have contributed to the reduction in biomass production of the plants grown in the VN soil. This can be attributed to the high concentration of metals, which when they exceed the plant’s tolerance level can cause a reduction in photosynthetic pigments and, consequently, photosynthetic capacity and growth (Suman et al. 2018; Schwalbert et al. 2022; Silva et al. 2022; Thiesen et al. 2023). This is because excess metals such as Cu, Zn, and Mn can inhibit the synthesis of chlorophyll or degrade it by replacing the Mg atom in the central structure of chlorophyll, which can lead to the degradation of the entire photosystem (He et al. 2022; Schwalbert et al. 2022; Küpper and Andresen 2016). In addition, the high concentration of Cu and Zn can lead to a reduction in the concentration of other essential ions in the shoot, such as Fe, and thus favor a reduction in chlorophyll content, which may have happened in *P. notatum* (Küpper and Andresen 2016; supplementary material, Table S3), but oxidative stress can also help to reduce the concentration of photosynthetic pigments (Dao and Beardall 2016; Küpper and Andresen 2016). A reduction in the concentration of photosynthetic pigments has already been observed in species of the Poaceae family when grown with high isolated levels of Cu (Silva et al. 2022), Zn (Schwalbert et al. 2022), and Mn (He et al. 2022; Thiesen et al. 2023).

The concentration of H_2_O_2_ observed in the leaves of *P. plicatulum* was lower when grown in VN soil compared to NF. This may be due to the increased activity of the SOD and POD enzymes in the leaves of this species (Fig. 5c and e; Gill and Tuteja 2010; Silva et al. 2022; Marques et al. 2023). In the antioxidant defense system, superoxide (O_2_•^−^), which is normally the first ROS to be generated, can be dismutated by SOD into H_2_O_2_ (Hasanuzzaman et al. 2020). Thus, SOD is the first line of defense against ROS, and SOD activity indicates that O_2_•^−^ is being degraded inside the cell and production of H_2_O_2_ occurs (Gill and Tuteja 2010). POD can catalyze oxidative reactions in plants, using H_2_O_2_ as a substrate (Gill and Tuteja 2010; Marques et al. 2023). Thus, our data suggest that the activity of the SOD and POD enzymes played an active role in combating oxidative stress caused by excess metals in *P. plicatulum* leaves. Thus, as observed in our study, increased activity of antioxidant enzymes such as SOD and POD has already been observed in grass species grown in Cu-contaminated soil under controlled conditions (Silva et al. 2022; Marques et al. 2023). In the roots of *P. plicatulum*, there was no change in the concentration of H_2_O_2_ when grown in VN and NF soil. In addition, lower activity of the enzymes SOD and POD was observed when grown in the VN soil. This may have been because there was no increase in the accumulated amount of Cu, Zn, and Mn in the roots of this species, and so it was not necessary to activate the antioxidant system in this region. In addition, as the metals were retained mainly in the cell wall and vacuoles, this may have contributed to lower toxicity and oxidative stress in this organ.

The concentration of H_2_O_2_ in the leaves of *P. notatum* was lower when grown in VN soil compared to NF. This can be attributed to a plant antioxidant defense system, enzymatic or non-enzymatic, which is efficient at combating ROS (Gill and Tuteja 2010). Other enzymes, besides POD, can also decrease the concentration of H_2_O_2_ (Gill and Tuteja 2010). This may explain the decrease in the concentration of H_2_O_2_ in *P. notatum* leaves without altering the activity of the POD enzyme. The concentrations of H_2_O_2_ in *A. compressus* leaves were also lower when grown in the VN soil, compared to the NF, which may mean an increase in other ROS. This is because other ROS can be formed through H_2_O_2_, including more reactive ones, such as the hydroxyl radical (OH^•^) (Gill and Tuteja 2010). This may explain the reduction in the concentration of photosynthetic pigments and leaf dry mass in this species, indicating greater stress (Küpper and Andresen 2016). On the other hand, the concentration of H_2_O_2_ was higher in the roots of the *A. compressus* and *P. notatum* species when grown in the VN soil. In addition, the activity of the SOD enzyme, but also the POD enzyme, in *A. compressus* was higher when grown in the VN soil. These results indicate that the antioxidant defense system in the roots of these species was not efficient, because even so, the concentration of H_2_O_2_ increased (Gill and Tuteja 2010).

### Phytostabilization potential

The high BCF values for Cu, Zn, and Mn in the species *A. compressus*, *P. plicatulum*, and *P. notatum*, when cultivated in the VN soil, suggest that these species can be used for phytostabilization of vineyards with more than 40 years of cultivation and high levels of Cu, Zn, and Mn in the soil (Silva et al. 2022). The species *P. plicatulum* and *P. notatum* accumulated higher concentrations of Zn and/or Mn in the shoot, but the concentrations were well below the classification to be considered hyperaccumulators (Baker and Brooks 1989).

*A. compressus* is the most sensitive species to the simultaneous increase in Cu, Zn, and Mn levels in the soil. This is because it was the species with the lowest TI (supplementary material, Table S2). This was due to the lower dry mass production of the shoot, caused by variations in nutritional status, a decrease in the concentration of photosynthetic pigments, and oxidative stress. Silva et al. (2022) observed that another species of the *Axonopus* genus was also sensitive to excess Cu, with doses of 70 mg Cu kg^−1^ in the soil causing plant death.

The *P. plicatulum* and *P. notatum* species showed higher TI, which indicates that these species have more efficient tolerance mechanisms to detoxify metals (Yan et al. 2020). This was observed in the present study, through the lower translocation of heavy metals to the leaves, compartmentalization of metals in metabolically less active subcellular areas, associated with a more efficient antioxidant defense system in the fight against ROS (Yan et al. 2020). However, even so, there was a reduction in the dry mass of the shoot in these species. However, we did not observe any change in the dry mass of the roots, an important organ for the phytostabilization of metals (Yan et al. 2020). This is because, in the process of phytostabilization, roots are fundamental for immobilizing heavy metals, stabilizing aggregates, and preventing soil erosion. It is therefore important for species to have a dense root system (Yan et al. 2020). Our study shows that enriching the vineyard with the species *P. plicatulum* and *P. notatum* can reduce the availability of metals.

## Conclusions

In vineyard soils with high Cu, Zn, and Mn contents (42.60 mg Cu dm⁻^3^, 17.37 mg Zn dm⁻^3^, and 162,54 mg Mn dm⁻^3^), a higher accumulation of these metals was generally observed in the leaves, stems, and roots of the three species evaluated. Exceptions were noted in the leaves and roots of *P. plicatulum*, the roots of *A. compressus*, and the stem of *P. notatum*, where Mn accumulation was lower.

The accumulation of Mn was the main variable explaining the variation in shoot dry mass of plants grown in VN and NF soils, due to its higher translocation to the aerial parts compared to Cu and Zn.

High concentrations of Cu, Zn, and Mn in the tissue generally cause variations in nutritional status, a decrease in chlorophyll, and increase the oxidative stress in the roots, which is reflected in lower dry mass production in the shoot of *A. compressus*,* P. notatum*, and *P. plicatulum*.

The species *A. compressus*,* P. notatum*, and *P. plicatulum* use mechanisms to tolerate excess Cu, Zn, and Mn, such as accumulation of the metals in the stem and/or roots, compartmentalization of the metals in metabolically less active areas, such as the cell wall and vacuole, and increased activity of the antioxidant enzymes SOD and POD.

*P. plicatulum* and *P. notatum* are the most suitable for phytostabilizing Cu, Zn, and Mn in vineyards with high levels of these metals in the Pampa biome.

## Supplementary Information

Below is the link to the electronic supplementary material.

ESM 1 (2.00 MB DOCX)

## Data Availability

All data supporting the findings of this study are available within the paper and its Supplementary Information.
