# Supplementary material for: Phytostabilization potential and tolerance mechanisms of native species from the Pampa biome in vineyard soil with high levels of Cu, Zn and Mn
Source: Environ Sci Pollut Res Int. 2026 Feb 2;33(7):2727–43. doi: 10.1007/s11356-026-37426-3 (PMC12982220; doi:10.1007/s11356-026-37426-3)
Supplement: Supplementary file 1 — (2.00 MB DOCX) [file 11356_2026_37426_MOESM1_ESM.docx]

**Environmental Science and Pollution Research**

**Phytostabilization potential and tolerance mechanisms of native species from the Pampa biome in vineyard soil with high levels of Cu, Zn and Mn**

Letícia Morsch^1^, Anderson Cesar Ramos Marques^2^, Edicarla Trentin^3^, Talita Andreolli^4^, Filipe Nunes de Oliveira^5^, Matheus Martins Ferreira^6^, Jean Michel Moura-Bueno^7^, Douglas Luiz Grando^8^, Adriele Tassinari^9^, Luciane Almeri Tabaldi^10^, Jucinei José Comin^11^, Arcângelo Loss^12^, Cledimar Rogério Lourenzi^13^, Gustavo Brunetto^14^

1 Postgraduate Program in Agroecosystems, Center of Agricultural Science, Universidade Federal de Santa Catarina (UFSC), 88034.001, Florianópolis, SC, Brazil. E-mail: lehmorsch@hotmail.com

2 Department of Biology, Universidade Federal de Santa Maria (UFSM), 97105-900, Santa Maria, RS, Brazil. E-mail: acrmarques@hotmail.com.br

3 Department of Soil Science, Universidade Federal de Santa Maria (UFSM), 97105-900, Santa Maria, RS, Brazil. E-mail: edicarlatrentin@gmail.com

4 Department of Soil Science, Universidade Federal de Santa Maria (UFSM), 97105-900, Santa Maria, RS, Brazil. E-mail: [talita.andreolli@acad.ufsm.br](mailto:talita.andreolli@acad.ufsm.br)

5 Department of Soil Science, Universidade Federal de Santa Maria (UFSM), 97105-900, Santa Maria, RS, Brazil. E-mail: aajvfilipe@hotmail.com

6 Center of Agricultural Science, Instituto Federal de Rondônia (IFRO) and Centro Universitário Faema (UNIFAEMA), Ariquemes, RO, Brazil. E-mail: math.ferreira10@yahoo.com.br

7 Department of Soil Science, Universidade Federal de Santa Maria (UFSM), Santa Maria, RS, Brazil and Universidade de Cruz Alta (UNICRUZ). E-mail: bueno.jean1@gmail.com

8 Department of Soil Science, Universidade Federal de Santa Maria (UFSM), 97105-900, Santa Maria, RS, Brazil. E-mail: douglas.agn@hotmail.com

9 Department of Soil Science, Universidade Federal de Santa Maria (UFSM), 97105-900, Santa Maria, RS, Brazil. E-mail: tassinaridrica@gmail.com

10 Department of Biology, Universidade Federal de Santa Maria (UFSM), 97105-900, Santa Maria, RS, Brazil. E-mail: lutabaldi@yahoo.com.br

11 Rural Engineering Department, Universidade Federal de Santa Catarina (UFSC), 88034.001, Florianópolis, SC, Brazil. E-mail: j.comin@ufsc.br

12 Rural Engineering Department, Universidade Federal de Santa Catarina (UFSC), 88034.001, Florianópolis, SC, Brazil. E-mail: arcangelo.loss@ufsc.br

13 Rural Engineering Department, Universidade Federal de Santa Catarina (UFSC), 88034.001, Florianópolis, SC, Brazil. E-mail: lourenzicr@gmail.com

14 Department of Soil Science, Universidade Federal de Santa Maria (UFSM), 97105-900, Santa Maria, RS, Brazil. E-mail: [brunetto.gustavo@gmail.com](mailto:brunetto.gustavo@gmail.com)

**Supplementary Material**


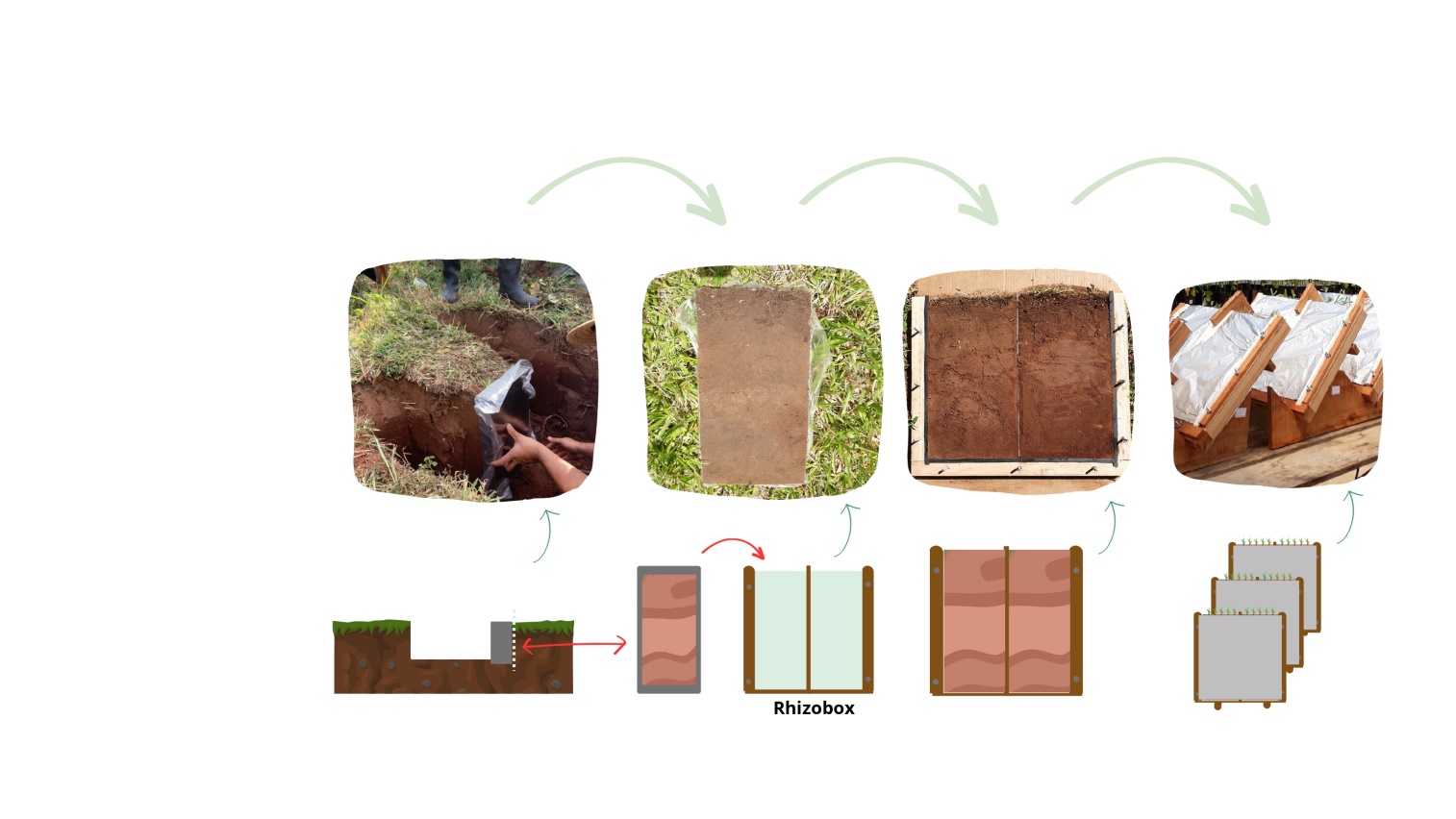


(a)

(b)

(c)

(d)

**Figure S1.** Diagram of how the soil was collected for the experiment.

**Figure S2.** Overview of rhizobox on greenhouse countertops (a). Overview of the rhizobox at the end of the experiment (b).

**Table S1.** Total Cu, Zn and Mn content accumulated in the tissue of *P. plicatulum*, *P. notatum* and *A. compressus*.

|  | Cu (µg rhizobox ^-1^) | | Zn (µg rhizobox ^-1^) | | Mn (µg rhizobox ^-1^) |
| --- | --- | --- | --- | --- | --- |
|  | NF | VN | NF | VN | ^(1)^ns |
| *A.* *compressus* | 1164.09aA^(2)^ | 1242.42aA | 679.44bC | 1345.03aC | 1794.85C |
| *P. notatum* | 429.45bB | 935.55aB | 1344.97bB | 1957.13aB | 2982.39B |
| *P. plicatulum* | 523.86bB | 973.03aB | 1583.41bA | 3924.31aA | 5036.71A |

^(1)^ns non-significant interaction;^(2)^ Capital letters compare species within each area and lower case letters compare species between areas. Equal letters do not differ statistically by the Scott-Knott test at 5% (P <0.05).

**Figure S3.** Distribution (%) of Cu (a), Zn (b) and Mn (c) accumulated in leaves, stems and roots of the species *A. compressus*, *P. notatum* and *P. plicatulum*, grown in vineyard soil (VN) and native field (NF).

**Table S2.** Translocation factor (TF), bioconcentration factor (BCF) of Cu, Zn and Mn, and tolerance index (TI) for plants grown in the vineyard soil.

|  | FT | | |  | BCF | | |  | TI |
| --- | --- | --- | --- | --- | --- | --- | --- | --- | --- |
|  | Cu | Zn | Mn |  | Cu | Zn | Mn |  | - |
| *A.* *compressus* | 0.05 | 0.31 | 0.49 |  | 12.46 | 26.50 | 2.31 |  | 0.73 |
| *P.notatum* | 0.21 | 1.39 | 2.86 |  | 4.62 | 12.05 | 1.26 |  | 0.95 |
| *P.plicatulum* | 0.16 | 0.97 | 2.51 |  | 6.65 | 23.20 | 1.83 |  | 0.85 |

**Figure S4.** Distribution (%) of Cu (a), Zn (b) and Mn (c) in the subcellular fractions, cell wall, nuclei and plastids, mitochondria and soluble fraction in leaves and roots of the species *A. compressus*, *P. notatum* and *P. plicatulum,* grown in vineyard soil (VN) and native field (NF).

**Table S3.** Nutrient content in organs of *A. compressus*, *P. notatum* and *P. plicatulum*.

| Species | Treatments | P | | | | K | Ca | Mg | Cu | Zn | Mn | Fe |
| --- | --- | --- | --- | --- | --- | --- | --- | --- | --- | --- | --- | --- |
|  |  | Leaf (g kg^-1^) | | | | | | | Leaf (mg kg^-1^) | | | |
| *A. compressus* | NF | 1.22bB^(1)^ | | | 9.30aB | | 2.74bC | 2.18bB | 6.75bB | 29.75bB | 42.19bC | 159.37aA |
|  | VN | 1.79aA | | | 9.11aA | | 4.78aB | 5.30aA | 12.50aA | 52.91aA | 121.05aB | 188.18aA |
| *P. notatum* | NF | 1.51aA | | | 11.39aA | | 3.92bB | 2.86bB | 12.01aA | 41.03bA | 315.36bB | 191.03aA |
|  | VN | 1.49aB | | | 7.19bB | | 8.50aA | 5.29aA | 11.36aA | 47.92aB | 461.21aA | 145.39bB |
| *P. plicatulum* | NF | 1.20aB | | | 8.18bB | | 5.72aA | 3.54aA | 11.22aA | 25.41bB | 688.84aA | 173.23aA |
|  | VN | 1.39aB | | | 10.02aA | | 3.60bC | 2.55bB | 7.13bB | 35.33aC | 474.54bA | 145.07aB |
| Stem (g kg^-1^) | | | | | | | | | Stem (mg kg^-1^) | | | |
| *A. compressus* | NF | | 0.77aB | | 4.43bC | | 1.04bB | 1.26bB | 3.95aB | 26.40bB | 59.71aC | 35.37aB |
|  | VN | | 1.09bB | | 6.86aB | | 1.41aB | 1.63aB | 7.58aC | 41.92aC | 43.61aB | 47.09aC |
| *P. notatum* | NF | | 1.23aA | | 8.18bB | | 1.05aB | 1.39bB | 11.35bA | 105.93bA | 185.38aB | 65.01aA |
|  | VN | | 1.47bA | | 10.17aA | | 0.97aC | 1.97aB | 20.62aB | 140.81aB | 72.06bB | 90.21aB |
| *P. plicatulum* | NF | | 1.18aA | | 9.61aA | | 2.04bA | 2.06bA | 11.58bA | 108.50bA | 233.13aA | 92.90bA |
|  | VN | | 1.63bA | | 9.96aA | | 2.74aA | 3.05aA | 29.76aA | 219.67aA | 201.33aA | 237.18aA |
| Root (g kg^-1^) | | | | | | | | | Root (mg kg^-1^) | | | |
| *A. compressus* | NF | | | 0.70bA | | 3.54bB | 1.70bA | 1.14bA | 134.48bA | 52.54bB | 185.38aA | 498.18bB |
|  | VN | | | 0.80aA | | 6.43aB | 2.80aA | 2.91aB | 217.81aA | 153.19aA | 169.82aA | 773.81aA |
| *P. notatum* | NF | | | 0.73bA | | 7.46bA | 1.55bA | 1.05bA | 76.14aB | 39.95bB | 127.74aB | 466.42aB |
|  | VN | | | 0.80aA | | 8.35aA | 2.26aB | 1.99aC | 80.86aC | 69.64aB | 92.98aB | 634.61aA |
| *P. plicatulum* | NF | | | 0.75bA | | 7.91bA | 1.88aA | 1.32bA | 72.78bB | 90.10bA | 182.92aA | 989.32aA |
|  | VN | | | 0.77aA | | 8.98aA | 2.06aB | 3.33aA | 116.22aB | 134.12aA | 134.54aA | 672.09bA |

^(1)^Capital letters compare species within each area and lower case letters compare species between areas. Equal letters do not differ statistically by the Scott-Knott test at 5% (P <0.05).
